# A Case of Refractory Peritoneal Dialysis-Related Peritonitis Caused by Streptococcus gordonii

**DOI:** 10.7759/cureus.77248

**Published:** 2025-01-10

**Authors:** Kenta Torigoe, Yoshie Yamashita, Ayuko Yamashita, Yuki Ota, Mineaki Kitamura, Naofumi Sakimura, Tadashi Uramatsu, Takahiro Takazono, Noriho Sakamoto, Kumiko Muta, Hiroshi Mukae, Tomoya Nishino

**Affiliations:** 1 Department of Nephrology, Nagasaki University Hospital, Nagasaki, JPN; 2 Respiratory Medicine, Nagasaki University Graduate School of Biomedical Sciences, Nagasaki, JPN

**Keywords:** antibiotic therapy, end-stage renal disease (esrd), pd peritonitis, peritoneal dialysis (pd), streptococcus gordonii

## Abstract

An 82-year-old woman undergoing peritoneal dialysis (PD) presented to our hospital with cloudy PD effluent since four days. The leukocyte count in the effluent was elevated to 2900/μL, and the patient was diagnosed with PD-related peritonitis. Treatment with cefazolin and ceftazidime was initiated; however, the leukocyte count in the effluent did not improve. Culture of the effluent revealed the presence of *Streptococcus gordonii*. Treatment was switched to ampicillin monotherapy; however, when the leukocyte count in the effluent still did not normalize, the PD catheter was removed for management of refractory PD-related peritonitis, which was then cured. *S. gordonii* can form biofilms, suggesting that PD-related peritonitis may develop through the formation of biofilms on the catheter, leading to refractory peritonitis.

## Introduction

Peritoneal dialysis (PD)-related peritonitis is a significant complication associated with increased mortality and the potential need to discontinue PD [1]. Gram-positive cocci are the most common pathogens causing PD-related peritonitis, comprising 39% of cases, though gram-negative rods and culture-negative infections are also observed [2]. Optimal treatment requires selection of the most efficacious antibiotics based on the causative organisms. Additionally, removing the PD catheter without delay is crucial when infections are caused by treatment-resistant organisms. Therefore, it is vital to gather comprehensive clinical data on PD-related peritonitis across various pathogens.

*Streptococcus gordonii*, a gram-positive facultative anaerobic coccus, is a common indigenous bacterium found on the skin, mouth, and intestine [3]. It has also been suggested to be involved in opportunistic infections such as bacterial endocarditis [4]. There are limited case reports of PD-related peritonitis due to *S. gordonii*; however, all cases were successfully treated without the need to remove the PD catheter [5-7].

We herein report a case of PD-related peritonitis caused by *S. gordonii*. Contrary to previous cases, removal of the PD catheter was required in this case to treat the refractory peritonitis.

## Case presentation

**Figure 1:**
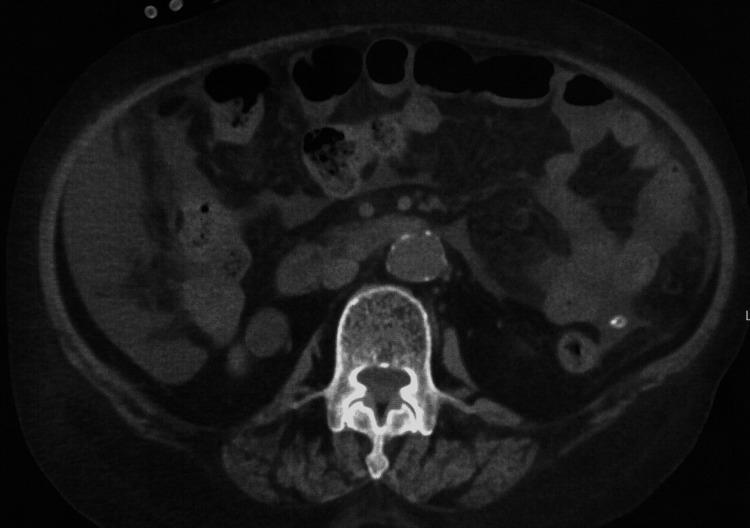
Computed tomography on admission showed no significant abnormalities other than the presence of a PD catheter and fluid. PD, peritoneal dialysis

An 82-year-old Japanese woman with end-stage renal disease due to nephrosclerosis had been undergoing PD for four years. The patient had no history of PD-related peritonitis. Four days before admission, she noticed cloudy PD effluent but had no symptoms and did not visit our department. On the scheduled outpatient visit day, the cloudiness in the PD effluent persisted. The WBC count in the PD effluent was 2900/μL (83% neutrophils), leading to a diagnosis of PD-related peritonitis. The patient was admitted to our department for treatment. She was 150.1 cm tall and weighed 54.1 kg. Her vital signs were as follows: body temperature, 36.3°C; blood pressure, 156/79 mmHg; pulse, 72/minute; and oxygen saturation, 99% (room air). Physical examination revealed no abdominal tenderness or signs of infection at the exit site or the tunnel of the PD catheter. Blood tests showed a mildly elevated CRP level (0.84 mg/dL), with no other significant findings. Plain abdominal computed tomography (CT) showed no evidence of intra-abdominal abscesses, enteritis, or other causes of peritonitis (Figure 1).

**Figure 2:**
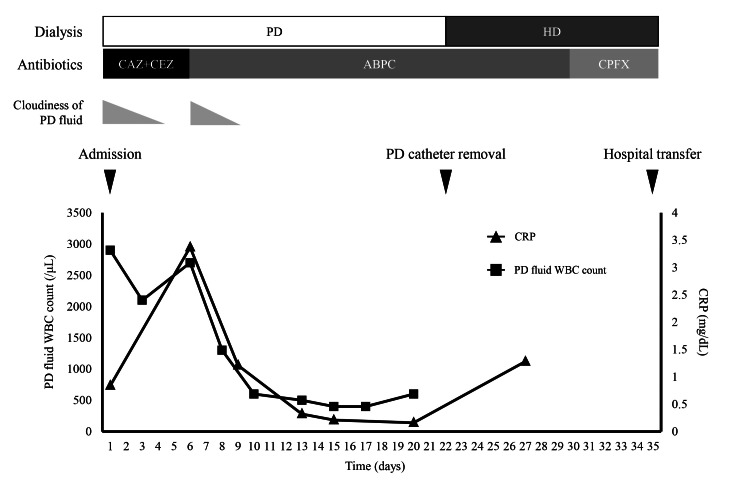
Clinical course of the patient ABPC, ampicillin; CAZ, ceftazidime; CEZ, cefazolin; CPFX, ciprofloxacin; CRP, C-reactive protein; HD, hemodialysis; PD, peritoneal dialysis; WBC, white blood cell

Following the International Society for Peritoneal Dialysis (ISPD) guidelines [8], intraperitoneal administration of cefazolin (1 g/day) and ceftazidime (1 g/day) was initiated (Figure 2).

After the treatment began, the cloudy effluent improved, and the WBC count in the PD effluent decreased to 2000/μL by hospital day 3. However, on day 6, the cloudy effluent reappeared, and the WBC count increased to 2700/μL. The initial culture of the PD effluent revealed the presence of *S. gordonii*. Based on the antimicrobial susceptibility profile, the antibiotic therapy was changed to intraperitoneal ampicillin sodium (1 g/day), which resolved the cloudiness (Table 1).

**Table 1:** Drug susceptibility test results for Streptococcus gordonii MIC, minimum inhibitory concentration

Antimicrobial agent	MIC (mg/mL)
Penicillin G	≦ 0.015
Ampicillin	≦ 0.5
Sulbactam/Ampicillin	≦ 0.5
Cefotaxime	≦ 0.5
Ceftriaxone	≦ 0.5
Cefepime	≦ 0.5
Imipenem/Cilastatin	≦ 0.12
Meropenem	≦ 0.06
Erythromycin	≦ 0.12
Clarithromycin	≦ 0.5
Clindamycin	≦ 0.12
Vancomycin	0.5
Levofloxacin	≦ 0.5
Linezolid	≦ 1
Daptomycin	0.5

The WBC count in the PD effluent decreased to 400/μL by day 17 but did not normalize, rising again to 600/μL by day 20. Repeat plain abdominal CT revealed no evidence of intra-abdominal abscesses or other pathological findings. The patient was diagnosed with refractory PD-related peritonitis, and the PD catheter was removed on hospital day 23. Culture of the catheter tip was negative. After the catheter was removed, the dialysis modality was changed from PD to hemodialysis (HD). Ampicillin sodium was administered intravenously, followed by oral ciprofloxacin, which was continued until day 35 of hospitalization. As the patient did not wish to return to PD, she was transferred to another hospital on day 35 for arteriovenous fistula creation and rehabilitation.

## Discussion

This case involved PD-related peritonitis caused by *S. gordonii*. Contrary to previous cases, the PD-related peritonitis was refractory and required removal of the PD catheter.

*S. gordonii* is a normal bacterium of the oral cavity; however, it is also involved in the development of apical periodontitis by adhering to dentin surfaces and forming biofilms. It also invades the bloodstream through dental caries and causes infective endocarditis by attaching to heart valves and forming biofilms. There are limited reports of PD-related peritonitis caused by *S. gordonii*, and only three cases have been reported to date [5-7].

Table 2 presents cases of *S. gordonii*-induced PD-related peritonitis, including the present case. Previous reports have shown that PD-related peritonitis caused by *S. gordonii* can be treated without removing the PD catheter. However, in this case, the PD-related peritonitis became refractory, and the patient required removal of the PD catheter. After the removal of the PD catheter, the patient was switched to HD. The ISPD guidelines define refractory PD-related peritonitis as effluent cloudiness or a leukocyte count >0.1 × 109/L that persists even after five days of appropriate antibiotic therapy [8]. In this case, *S. gordonii* was identified in the PD effluent, and ampicillin was administered for more than five days according to the drug susceptibility test; however, the leukocyte count in the PD effluent did not normalize.

**Table 2:** Cases of PD-related peritonitis due to Streptococcus gordonii ESRD, end-stage renal disease; HD, hemodialysis; PD, peritoneal dialysis

References	Age/sex	Cause of ESRD	Duration of peritoneal dialysis	Clinical findings	Antibiotic treatment	PD catheter removal	Transfer to HD	Outcome
Cheung et al. [5]	69/Male	Chronic glomerulonephritis	3 years	Abdominal pain, cloudy peritoneal fluid	Cefazolin＋cefepime to cefazolin	No	No	Cured
Maraki et al. [6]	70/Female	Chronic pyelonephritis	12 years	Abdominal pain, cloudy peritoneal fluid, fever ,nausea	Vancomycin + amikacin to meropenem	No	No	Cured
Lim et al. [7]	54/Male	Unknown	1 year	Abdominal pain, cloudy peritoneal fluid	Ceftriaxone + cloxacillin to ampicillin	No	No	Cured
Current case	82/Female	Nephrosclerosis	4 years	Cloudy peritoneal fluid	Cefazolin＋ceftazidime to ampicillin to ciprofloxacin	Yes	Yes	Cured

Risk factors for refractory peritonitis have not been well established. However, some of the reported factors include long PD duration, female sex, and gram-negative bacilli [9]. Furthermore, biofilm formation on PD catheters has been reported to be a factor in refractory peritonitis [10]. Considering the biofilm-formation ability of *S. gordonii* in the oral cavity and heart valves, it is possible that *S. gordonii* formed a biofilm on the PD catheter in this case. However, when a culture test was performed on the tip of the PD catheter after it was removed in this case, no bacteria were detected. Although electron microscopy is effective in detecting biofilm formation on PD catheters [11], it was not performed in this case; therefore, it was not possible to conclude whether biofilm formation was involved. Considering the properties of *S. gordonii*, biofilm formation should be considered when PD-related peritonitis is refractory. In patients with refractory PD-related peritonitis, failure to remove the PD catheter in a timely manner is associated with increased hospital stay, peritoneal damage, increased risk of fungal peritonitis, and increased mortality [8]. Therefore, patients with PD-related peritonitis caused by *S. gordonii* should be treated carefully, with close attention being paid to the progression of the disease to refractory peritonitis.

## Conclusions

We reported a case of refractory PD-related peritonitis caused by *S. gordonii* that became refractory and the patient required catheter removal. Considering the biofilm-forming nature of *S. gordonii*, it is quite possible that PD-related peritonitis caused by *S. gordonii* can become refractory.
